# miR-383-5p, miR-181a-5p, and miR-181b-5p as Predictors of Response to First-Generation Somatostatin Receptor Ligands in Acromegaly

**DOI:** 10.3390/ijms24032875

**Published:** 2023-02-02

**Authors:** Daniel G. Henriques, Renan Lyra Miranda, Rômulo Sperduto Dezonne, Luiz Eduardo Wildemberg, Aline Helen da Silva Camacho, Leila Chimelli, Leandro Kasuki, Elisa B. Lamback, Alexandro Guterres, Monica R. Gadelha

**Affiliations:** 1 Endocrine Unit and Neuroendocrinology Research Center, Medical School and Hospital Universitário Clementino Fraga Filho, Universidade Federal do Rio de Janeiro, Rio de Janeiro 21941-617, Brazil; 2 Neuropathology and Molecular Genetics Laboratory, Instituto Estadual do Cérebro Paulo Niemeyer, Rio de Janeiro 20231-092, Brazil; 3 Neuroendocrinology Division, Instituto Estadual do Cérebro Paulo Niemeyer, Rio de Janeiro 20231-092, Brazil; 4 Endocrinology Division, Hospital Federal de Bonsucesso, Rio de Janeiro 21041-020, Brazil

**Keywords:** pituitary tumors, acromegaly, microRNA, biomarkers, response to fg-SRL

## Abstract

Acromegaly is a chronic systemic disease caused in the vast majority of cases by growth hormone (GH)-secreting adenoma, with surgery being the first-line treatment. When a cure is not attained with surgery, first-generation somatostatin receptor ligands (fg-SRLs) are the most common medication prescribed. Predictors of response to fg-SRLs have been studied; however, they cannot fully predict the response to fg-SRL. MicroRNAs are small RNAs, the main role of which is messenger RNA (mRNA) post-transcriptional regulation. This study aimed to identify the microRNAs involved in resistance to treatment with fg-SRLs in acromegaly. Ten patients with acromegaly undergoing treatment with fg-SRLs were selected to undergo miRNA sequencing: five controlled and five uncontrolled with treatment. Bioinformatic analysis was performed to detect differentially expressed miRNAs. Then, the same 10 samples were used for validation by qPCR and an additional 22 samples were analyzed, totaling 32 samples. e We found 59 differentially expressed miRNAs in the first analysis. miR-181a-5p and miR-181b-5p were downregulated, and miR-383-5p was upregulated in the uncontrolled group. Receiver operating characteristic (ROC) curve analysis of miR-383-5p showed an NPV of 84.3% and a PPV of 84.5%. In summary, miR-181a-5p, miR-181b-5p, and miR-383-5p are biomarkers of response to fg-SRLs, and they can be used individually or included in prediction models as tools to guide clinical decisions.

## 1. Introduction

Acromegaly is a rare systemic disease caused mainly by a sporadic growth hormone (GH)-secreting pituitary adenoma that leads to increased serum concentrations of insulin-like growth factor type I (IGF-I) [1]. Patients diagnosed with acromegaly have a higher mortality rate than the general population, if the disease is not adequately controlled [2,3]. Furthermore, it causes arterial hypertension, diabetes mellitus, arthralgia, and enlargement of the extremities, among other complications [2,3,4]. First-line treatment is surgery [5], attaining a cure in up to 80% of microadenomas and 40–70% of macroadenomas in specialized centers [6,7]. Patients who do not attain a cure after surgery receive pharmacological treatment and, in more aggressive cases, radiotherapy [5].

The first-generation somatostatin receptor ligands (fg-SRLs), octreotide and lanreotide, are the most commonly used medications to control GH and IGF-I levels in patients with acromegaly [6]. These medications act preferentially through somatostatin receptor type 2 (SST2), activating an inhibitory G protein that inhibits cyclic AMP (cAMP) production by adenylyl cyclase [8]. Downstream, this signaling pathway inhibition leads to lower GH secretion, cell proliferation inhibition, and apoptosis [8,9].

Biochemical control is achieved in only 30–40% of patients with fg-SRLs [10,11]. For this reason, some efforts have been undertaken to predict which patients respond better to these drugs. Low levels of SST2, for instance, are associated with worse treatment response [12,13]. However, the expression of SST2 does not completely explain the resistance to fg-SRLs since some patients with high levels of SST2 do not respond to therapy [11]. Moreover, there have been substantial attempts to combine different tumor and clinical characteristics to build prediction models. Recently, our group developed a machine learning model that achieved high accuracy in predicting biochemical control after fg-SRL treatment [11]. However, there remains much to be understood regarding treatment resistance with fg-SRLs in acromegaly [14].

In recent decades, microRNAs (miRNAs) have been studied not only to understand tumorigenesis and improve diagnosis [15] but also to comprehend prognosis and response to treatment and to develop new potential therapies [16,17]. MiRNAs are small noncoding RNAs with an important role in post-transcriptional regulation, mainly as the most important suppressor of protein translation [18]. Therefore, some studies have analyzed the role of miRNAs in tumorigenesis and as biomarkers in acromegaly [19,20,21,22]. In past studies, miRNAs have already been correlated with poor fg-SRL response by acting directly or indirectly in the SST2 signaling pathway [19,20,22].

In the present study, we aimed to evaluate which miRNAs might act as accurate biomarkers of response to fg-SRL treatment and their associations with tumor characteristics.

## 2. Results

### 2.1. Cohort Selection and Differentially Expressed miRNAs in Uncontrolled Patients with fg-SRL Treatment

Among consecutive patients with acromegaly from our center, we selected 32 patients who had available data and frozen tumor samples. Our cohort was separated into two different groups according to disease control with fg-SRLs, controlled and uncontrolled (Table 1). In this cohort, we found a near statistical significance for age, sex, median IGF-I before treatment, granulation pattern, and SST2 IRS (Table 1). Since GH and IGF-I decreases are important to establish disease control, we also evaluated these two factors, and they were significantly different between the groups (*p* = 0.003 and *p* = 0.001, respectively).

From our initial cohort, we selected a subset of ten patients in whom miRNA sequencing by a next-generation platform was performed. Five patients were excellent responders (controlled), with GH < 1 ng/mL and normal IGF-I at re-evaluation, and five were very poor responders (uncontrolled), with GH > 1 ng/mL and a decrease in IGF-I of less than 50% (Table 2). Therefore, for this evaluation, we selected two very different groups according to their biochemical responses to fg-SRLs. We had available material to perform radiological analysis in four controlled and three uncontrolled patients and analysis of granulation pattern and somatostatin receptors in four controlled and four uncontrolled patients. As seen in Table 2, the controlled patients had very high GH and IGF-I decrease percentages during fg-SRL treatment, while the uncontrolled group even had increased GH and IGF-I levels. Furthermore, the controlled patients had a higher median age, lower pretreatment GH and IGF-I levels, smaller tumor diameters, and higher SST2 IRS. Moreover, a sparsely granulated pattern and invasive tumors were most prevalent in the uncontrolled group. The sample size was not powered for statistical analysis.

**Figure 1:**
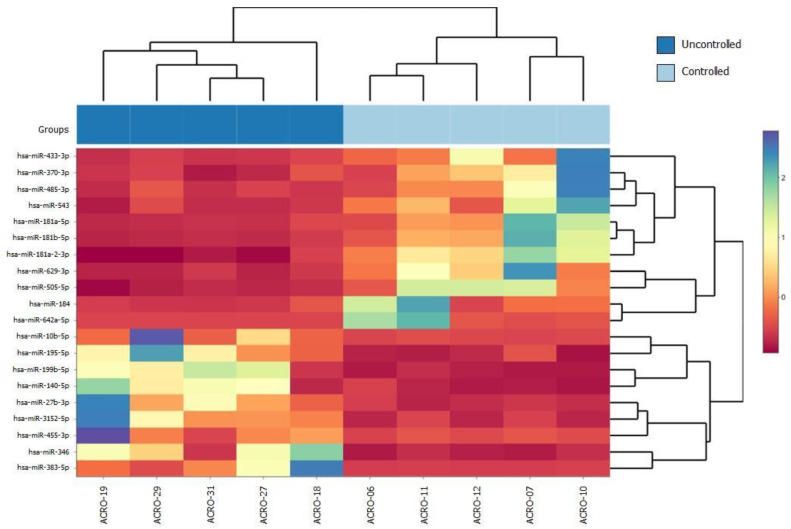
Heatmap of the top 20 differentially expressed miRNAs between the controlled and uncontrolled groups. The heatmap shows miRNA expression in each sample through the contrast of colors. On the left, the top 20 differentially expressed miRNA are depicted and clustered randomly according to their expression (clustering tree in the right); representative colors were used to identify expression (red is underexpressed; blue is overexpressed). On the bottom, the patients are displayed, and on the top, grouped samples appear according to their miRNA expression (light blue is controlled; dark blue is uncontrolled). hsa: Homo sapiens; miR: microRNA.

After miRNA sequencing, we performed a differential expression analysis according to the response to fg-SRLs using DESeq2. We found 59 differentially expressed miRNAs between the controlled and uncontrolled patients. The top 20 differentially expressed genes are listed in Figure 1. Hierarchical clustering of the top 20 differentially expressed miRNAs between the controlled and uncontrolled groups was performed to build a heatmap (Figure 1).

**Figure 2:**
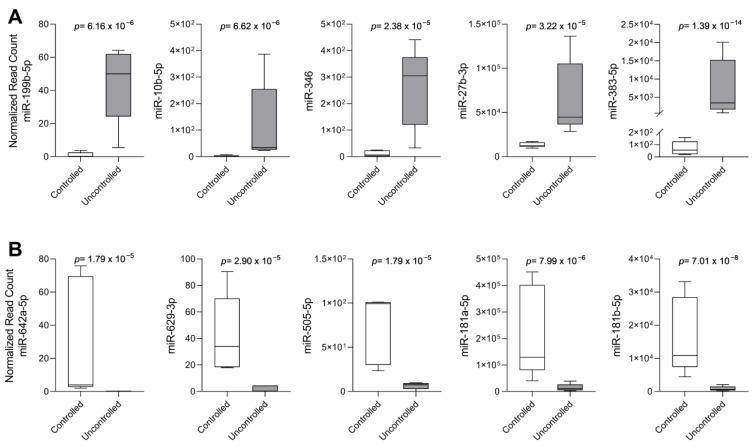
Top 10 differentially expressed miRNAs. Box plot graphs show the miRNAs’ expression according to their normalized read count in each group of patients. (**A**) Overexpressed miRNA in the uncontrolled group. (**B**) Underexpressed miRNA in the uncontrolled group.

We chose the top 10 differentially expressed miRNAs, five underexpressed and five overexpressed in the uncontrolled group, for the validation step due to their statistical significance in the DESeq2 analysis, with them all showing adjusted *p*-values < 0.00005. In the uncontrolled group compared to the controlled group, we found the following: the overexpressed miRNAs were miR-10b-5p, miR-27b-3p, miR-199b-5p, miR-346, and miR-383-5p (Figure 2A), while the underexpressed miRNAs in the same group were miR-181a-5p, miR-181b-5p, miR-505-5p, miR-629-3p, and miR-642a-5p (Figure 2B).

### 2.2. Analysis of the Top 10 Differentially Expressed miRNAs by qPCR

Initially, we performed technical validation of miRNA sequencing data by analyzing the top 10 differentially expressed miRNAs identified during miRNA sequencing through qPCR in the same samples. We found that six out of ten miRNAs were differentially expressed between the controlled and uncontrolled groups, thus reproducing the previous findings: miR-181a-5p (*p* = 0.0079) and miR-181b-5p (*p* = 0.0079) were upregulated in the controlled group; meanwhile, miR-383-5p (*p* = 0.0097), miR-199b-5p (*p* = 0.0195), miR-10b-5p (*p* = 0.0254), and miR-27b-3p (0.0317) were upregulated in the uncontrolled group. The expression of two miRNAs was not different between the groups by qPCR: miR-629-3p (*p* = 0.4633) and miR-505-5p (*p* = 0.8294). Furthermore, miR-642-5p and miR-346 were not amplified in any of the samples. Therefore, they were excluded from subsequent the analysis.

**Figure 3:**
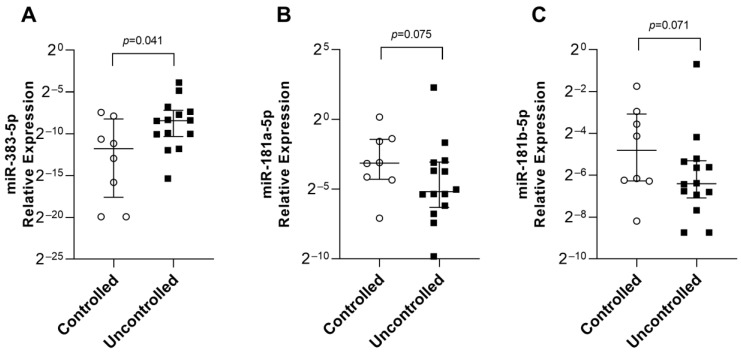
miRNAs differentially expressed between the controlled and uncontrolled groups according to their relative expression. (**A**) miR-383-5p. (**B**) miR-181a-5p. (**C**) miR-181b-5p. On the *y*-axis, the relative expression is scaled by Log2. On the *x*-axis are the controlled and uncontrolled groups. Black squares are uncontrolled tumors, and white dots are controlled tumors.

Afterward, we analyzed the eight miRNAs in the remaining 22 patients for biological validation, of whom eight were controlled and 14 were uncontrolled (Table 3). We had available material to perform radiological analysis in four controlled and 10 uncontrolled patients and analysis of granulation pattern and somatostatin receptors in five controlled and 12 uncontrolled patients. Our results showed that miR-383-5p was upregulated in the uncontrolled group (*p* = 0.041) (Figure 3A). Meanwhile, miR-181a-5p and miR-181b-5p were downregulated (*p* = 0.075 and *p* = 0.071, respectively) (Figure 3B,C). miR-10b-5p, miR-27b-3p, miR-199b-5p, miR-505-5p, and miR-629-3p expression between the groups was not different (Table 4).

### 2.3. miR-383-5p, miR-181a-5p, and miR-181b-5p as Predictors of Response to fg-SRL Treatment

We investigated miR-383-5p, miR-181a-5p, and miR-181b-5p’s ability to predict disease control, considering all 32 patients. miR-383-5p had a negative correlation with IGF-I decrease (R = −0.456; *p* < 0.01), while miR-181a-5p (R = 0.487; *p* < 0.01) and miR-181b-5p (R = 0.487; *p* < 0.01) demonstrated a positive correlation with IGF-I decrease. MiR-181b-5p (R= 0.396; *p* < 0.05) also showed a positive correlation with GH decrease.

**Figure 4:**
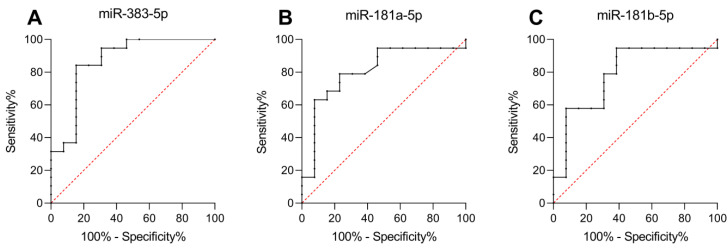
miR-383-5p, miR-181a-5p, and miR-181b-5p are predictors of response. ROC curve of differentially expressed miRNAs that correlated with responsiveness. (**A**) miR-383-5p ROC curve with *p* = 0.005. (**B**) miR-181a-5p ROC curve with *p* = 0.019. (**C**) miR-181b-5p ROC curve with *p* = 0.027.

A ROC curve was generated to assess whether the three miRNAs were good biomarkers for determining the response to treatment (Figure 4). miR-181a-5p (*p* = 0.019; AUC = 0.747; 95% CI: 0.570–0.924) had a sensitivity of 69.2%, a specificity of 73.7%, a negative predictive value (NPV) of 70.6%, and a positive predictive value (PPV) of 72.5%. Moreover, miR-181b-5p (*p* = 0.027; AUC = 0.733; 95% CI: 0.552–0.914) showed a sensitivity of 69.2%, a specificity of 63.2%, an NPV of 67.2%, and a PPV of 65.3%. Furthermore, miR-383-5p demonstrated an excellent ability to predict unresponsiveness (*p* = 0.005; AUC = 0.866; 95% CI: 0.727–1.000), with a sensitivity of 84.2%, a specificity of 84.6%, an NPV of 84.3%, and a PPV of 84.5%.

### 2.4. miR-383-5p, miR-181a-5p, and miR-181b-5p Are Associated with Tumor and Patient Characteristics

Initially, we evaluated the relationship between miRNAs’ expression and gender and age. There was no difference in the relative expression between males and females for the three miRNAs. Moreover, when analyzing the relative expression in the controlled versus uncontrolled groups taking into consideration only individuals of the same gender, there were still differences between the groups (Supplementary Figure S1). Additionally, when we correlated the miRNAs’ relative expression with patients’ age, we found no significant statistical correlation (miR-383-5p: R: −0.1598/*p* = 0.38; miR-181a-5p: R:0.14/*p* = 0.45; miR-181b-5p: R:0.1373/*p* = 0.45).

**Figure 5:**
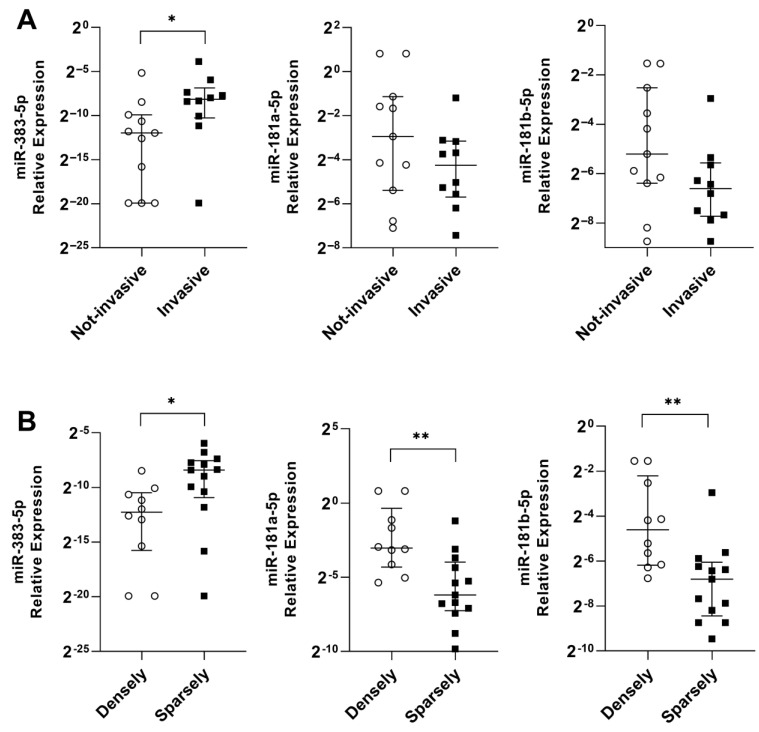
miR-383-5p, miR-181a-5p, and miR-181b-5p and tumor characteristics. (**A**) miRNAs’ relative expression in noninvasive x invasive tumors. (**B**) miRNAs’ relative expression in densely granulated tumors x sparsely granulated tumors. On the *y*-axis, the relative expression is scaled by Log2. *: *p* < 0.05; **: *p* < 0.005.

In sequence, we evaluated the associations between miRNA expression and tumor characteristics. miR-383-5p was overexpressed (*p* = 0.012), and both miR-181a-5p (*p* = 0.0021) and miR-181b-5p (*p* = 0.0025) were underexpressed in sparsely granulated tumors (Figure 5). Moreover, miR-383-5p was overexpressed in invasive tumors (*p* = 0.02), while miR-181a-5p and miR-181b-5p expression was not statistically significant between the groups (*p* = 0.25 and *p* = 0.10, respectively). We did not perform comparisons with SST2 since we had only four patients with low SST2 (IRS < 6).

## 3. Materials and Methods

### 3.1. Patients

This study was a retrospective single-center study performed at the Instituto Estadual do Cérebro Paulo Niemeyer and included 32 patients from up to ten years ago. It was registered with Plataforma Brasil (41010620.0.1001.8110), approved by the Institutional Review Board/Ethics Committee, and conducted in accordance with the Declaration of Helsinki. Patients signed informed consent forms prior to study entry.

The inclusion criteria consisted of the following: (1) patients older than 18 years old with acromegaly caused by GH-secreting pituitary adenoma confirmed by laboratory and histopathology exams; (2) surgical resection of the GH-secreting pituitary adenoma and availability of fresh frozen tumor samples for DNA and RNA extraction; and (3) treatment with fg-SRLs with data on the response. The exclusion criteria consisted of: (1) patients treated with fg-SRLs, pasireotide, dopamine agonists, pegvisomant, or radiotherapy prior to surgery; and (2) patients diagnosed with acromegaly associated with genetic diseases (multiple endocrine neoplasia type 1 or 4, familial isolated pituitary adenoma, Carney complex, 3PA syndrome, and McCune–Albright syndrome).

Demographic (age at diagnosis and gender), biochemical (GH and IGF-I levels at diagnosis before and after medical treatment with fg-SRL), and radiological data were collected. Cavernous sinus invasion was defined according to the modified Knosp–Steiner classification, with Knosp 3 or higher being classified as invasive [23]. GH and IGF-I levels were analyzed after 6 months of treatment with the maximum necessary fg-SRL dose (lanreotide Autogel 120 mg or octreotide LAR 30 mg). Controlled patients were defined as GH < 1.0 ng/mL and normal IGF-I adjusted for age. In this study, 13 patients were controlled and 19 were uncontrolled. From these patients, we had available material to perform radiological analysis in eight controlled and 13 uncontrolled patients; analysis of granulation pattern in nine controlled and 14 uncontrolled samples; and analysis of somatostatin receptors in nine controlled and 16 uncontrolled samples.

### 3.2. Immunohistochemistry

As previously described [24], immunohistochemistry was performed with GH [Research Resource Identifier (RRID): AB_1158348; dilution 1:6000], prolactin (RRID: AB_1158781; dilution 1:7000), a monoclonal α-subunit of glycoprotein hormones (RRID: AB_297871; dilution 1:6000), and estrogen receptor-alpha (RRID: AB_1661112; dilution 1:600) antibodies to confirm the diagnosis of acromegaly and classify tumors as pure or mixed somatotropinomas according to the new World Health Organization classification [25]. A cytokeratin antibody (CAM5.2) [RRID: AB_2891304, dilution 1:10,000] was used to define the granulation pattern and classify tumors as sparsely granulated (SG), densely granulated (DG), or negative [26]. Rabbit monoclonal antibodies against SST2 (RRID: AB_2737601, dilution 1:5000) and SST5 (RRID: AB_10859946, dilution 1:2000) were assessed using the immunoreactive score (IRS), and tumors were considered to present high expression when the IRS was greater than 5 [27]. The detection kit used was a Novolink™ Max polymer Detection System (RE7260-CE; Leica Biosystems, Deer Park, IL, USA). The samples were analyzed by two different observers (A.H.S.C. and L.E.W.), and discordant results were submitted for evaluation by a third observer (L.C.).

### 3.3. RNA Extraction

Isolation of RNA from 32 tumors was performed using an Allprep DNA/RNA/miRNA Universal Kit (Qiagen, Hilden, Germany), and the RNA samples were treated with DNase (Turbo DNA-free™ Kit, Invitrogen, Waltham, MA, USA) according to the manufacturer’s protocols. RNA concentration was obtained using a Qubit™ RNA Broad-Range Assay Kit (Invitrogen, Life Technologies, Carlsbad, CA, USA), and RNA purity was assessed by a NanoDrop Lite spectrophotometer (Thermo Fischer, Wilmington, DE, USA) with an optimum ratio between 1.9 and 2.1.

### 3.4. MicroRNA Sequencing (miRNA-seq)

Total RNA samples from 10 tumors were sent to the Beijing Genomics Institute Tech Solutions company (BGI, Shenzhen, China), where the RNA integrity was assessed by a 2100 bioanalyzer™ (Agilent Technologies, Inc., Santa Clara, CA, USA), and the RNA integrity number (RIN) was evaluated. For further analysis, we used only samples with a RIN value greater than 8.5. MiRNA enrichment and purification, library preparation, and sequencing were performed on the BGISEQ-500 platform according to their specifications. MiRNA-seq was performed by next-generation sequencing using combinatorial probe-anchor synthesis (c-PAS) and DNA nanoball (DNB™) sequencing technologies [28].

Data preprocessing was performed by BGI, which included adapter and barcode removal, resulting in more than 10 million clean reads for each sample. We aligned clean reads to 2652 human mature miRNAs from miRbase v22 [29] using the Bowtie algorithm, considering only reads longer than 17 bases with no mismatches. Unaligned reads were aligned with the miRNA hairpin sequence database, sRNA database, and tRNA database. We analyzed differentially expressed miRNAs between the controlled and uncontrolled groups using the DESeq2 algorithm [30] through sRNAtoolbox [31].

### 3.5. Quantitative Real-Time Polymerase Chain Reaction

Ten nanograms of total RNA samples were used to perform miRNA reverse transcription and amplification using a TaqMan™ Advanced miRNA cDNA Synthesis Kit (Life Technologies Corporation, Carlsbad, CA, USA) according to the manufacturer’s protocol. Steps that required thermocyclers were performed in a C1000 Touch™ thermal cycler (Applied Biosystems, Foster City, CA, USA). After this step, we diluted the miR-amplification products from 1 to 5. A quantitative polymerase chain reaction was performed in triplicate with TaqMan™ Advanced miRNA Assays (Life Technologies Corporation) and used 2.5 µL of the diluted miR-amplification product, with 5 µL of TaqMan^®^ Fast Advanced Master Mix (2×), 0.5 µL of TaqMan^®^ Advanced miRNA Assay (20×), and 2 µL of RNase-free water. Our group used the CFX96™ Touch Real-Time PCR Detection System (Applied Biosystems) with the following steps: 1 cycle of enzymatic activation at 95 °C for 20 s, followed by 40 cycles of denaturation at 95 °C for 1 s and annealing/extension at 60 °C for 20 s. Each assay was performed in triplicate for each sample. The miRNA assays were as follows: miR-10b-5p (assay ID: 478494_mir), miR-26a-5p (assay ID 477995_mir), miR-27b-3p (assay ID: 478270_mir), miR-181a-5p (assay ID: 477857_mir), miR-181b-5p (assay ID: 478583_mir), miR-199b-5p (assay ID: 478486_mir), miR-346 (assay ID: 478046_mir), miR-383-5p (assay ID: 478079_mir), miR-505-5p (assay ID: 478957_mir), miR-629-3p (assay ID: 479478_mir), and miR-642a-5p (assay ID: 479121_mir). miR-26a-5p was used as a reference since the manufacturer’s protocol suggested it as a reference miRNA, and in our samples, it was found to have the lowest normalized read variation in miRNA sequencing. To analyze the results for each sample, we obtained the mean cycle threshold (Ct) from replicates for each miRNA and subtracted the reference Ct, resulting in ∆Ct. Then, we calculated the relative expression with the formula 2^−∆Ct^ [32].

### 3.6. Statistical Analysis

Statistical analysis, graphs, and ROC curves were generated with Prism Graphpad Prism software, version 8.0 for Windows (Graphpad Software, San Diego, CA, USA), and SPSS statistics software, version 23.0 for Windows (IBM, Chicago, IL, USA). The Mann–Whitney U test and the chi-square and Fisher’s exact tests were performed for numerical and categorical variables, respectively. Correlation analysis was performed by Spearman’s test. The results were considered statistically significant when the *p*-value was < 0.05.

## 4. Discussion

This study aimed to investigate miRNAs as biomarkers of response to fg-SRLs. In our cohort, the patients and tumor characteristics were in accordance with previous studies, showing that it is representative of patients with acromegaly [11,12,27,33,34]. Our study described three new miRNAs associated with the response to fg-SRL and tumor characteristics. We found that miR-181a-5p and miR-181b-5p were downregulated in the fg-SRL uncontrolled group and were underexpressed in sparsely granulated tumors. Additionally, miR-383-5p was upregulated in the uncontrolled group and was overexpressed in invasive tumors and sparsely granulated tumors. Finally, we demonstrated that the miRNA expression levels of miR-383-5p, miR-181a-5p, and miR-181b-5p were not correlated to age and gender. 

Our study has fundamental differences in methodology and patient selection compared to previous studies, which influenced our results. Our patients did not initiate any medication prior to surgery, and the biochemical control was defined as in recent guidelines [5]. For example, Mao et al.’s [22] study differed from ours since they performed a microarray in some acromegaly patients who had initiated fg-SRLs before surgery, which might have altered miRNA expression in their samples, and their response definition was GH decrease alone [22]. Furthermore, we selected two very different groups: very good responders in the controlled group and resistant patients in the uncontrolled group, which reflected GH and IGF-I percentage variations. Other studies have mainly tested miRNAs by investigating their targets, which was not our main goal [19,20,35].

Our study demonstrated that miR-181a-5p and miR-181b-5p are biomarkers of response to fg-SRL treatment. The miR-181 family acts in different neoplasias as a tumor suppressor and oncomiR in cancers [36,37]. One of the pathways that is influenced by the miR-181 family is the mitogen-activated protein kinase (MAPK) pathway [37]. This pathway is downstream of SST2 signaling and is downregulated to achieve reduced hormone secretion and proliferation [8,38]. miR-181a-5p directly targets the 3′UTR of Ras family protein mRNA, specifically downregulating MAPK in acute myeloid leukemia cells [39].

It was also shown that miR-181a-5p regulates c-AMP response element-binding protein (CREB)-1 and might also participate in a feedback loop with CREB1-regulating miR-181a-5p in bone fibrous dysplasia [40]. Therefore, since SST signaling acts to suppress the MAPK pathway and CREB, upregulation of miR-181a-5p might maintain low basal levels of proteins in this signaling pathway, with it being a biomarker of a better response. However, both miR-181a-5p and miR-181b-5p might act as oncomiRs. In colorectal cancer, miR-181a-5p binds to PTEN (phosphatase and tensin homolog) mRNA [41], an antagonist of the PI3K-AKT pathway that is related to cell growth, protein synthesis, and proliferation [42]. An in vitro study that used A549 cells, i.e., adenocarcinomic human alveolar basal epithelial cells, showed an inverse correlation between miR-181a-5p and E-cadherin, suggesting the participation of this miRNA in epithelial–mesenchymal transition [43].

MiR-383-5p was found to be an excellent biomarker, with it being overexpressed in uncontrolled patients with good PPV and NPV. However, this result is different from most data about this miRNA, which has been primarily described as a tumor suppressor [44]. This miRNA was described as being downregulated in invasive nonfunctioning pituitary adenomas [45]. However, there have been some reports demonstrating miR-383-5p as an oncomiR, which might be explained by its role in regulating the proteins involved in apoptosis [44]. For instance, miR-383-5p targets the protein caspase-2 [46], which plays a major role in the mitochondrial apoptotic pathway and in the p53 activation pathway [47]. It has also been demonstrated that miR-383-5p protects rat neuron cells against apoptosis by inhibiting the Bax/Bcl-2 signaling pathway [48]. According to the miRDB database, another possible target of miR-383-5p in inhibiting apoptosis is PERP (TP53 apoptosis effector), which has p53 binding elements that are all occupied under apoptotic conditions [49]. However, further in vivo analysis must be considered to confirm the correlation between PERP and miR-383-5p. Therefore, miR-383-5p plays a major role in apoptosis, and its overexpression might impair this mechanism in somatotropinoma cells, decreasing the ability of fg-SRLs to induce cell death. Hence, its overexpression might make the tumor more resistant to treatment.

Discovering good biomarkers of response to fg-SRL is important in acromegaly treatment, not only to have more accuracy in precision medicine [14] but also for the rational use of medication since treatment with fg-SRL can cost USD 7195.00 to USD 15,970.00 annually per patient in Brazil [50]. To date, the literature has shown some factors that predict the response to fg-SRLs with good accuracy. For instance, SST2 has also been found in high levels in control patients [12,27,51], which can be corroborated by our study, which positively correlated IGF-I and GH decreases with SST2 IRS (data not shown). However, high SST2 does not guarantee a good response, and other factors influence the treatment response. Some of these characteristics were combined in a machine learning algorithm created by our group in an attempt to obtain a better prediction value [12]. The model with the best performance included the following features: age, sex, pretreatment IGF-I and GH levels, SST2 IRS, SST5 IRS, and granulation pattern; the model achieved an accuracy of 86.3%, an NPV of 87.5%, and a PPV of 83.3% [11]. E-cadherin has also been tested as a biomarker with a good response with an NPV of 81.3% and a PPV of 100% [13]. Combining more tools in the decision-making process is a challenge since more biomarkers must be discovered to characterize a phenotypic pattern for good responders, and miRNA expression can help in this prediction.

The present study was performed in a highly selected sample representative of the acromegaly patient population, without confounding factors, such as pretreatment with medical therapy. However, it has some limitations. Despite our sample being quite representative, due to the strict selection criteria, the sample was created from a small group of patients. For this reason, we chose ten samples to perform miRNA sequencing as a subset of our cohort. However, this subpopulation represented the controlled and uncontrolled phenotypes well since it was composed of the best and worst responders to treatment. Nonetheless, we cannot exclude the possibility of other microRNAs emerging as differentially expressed in a larger sample. However, this fact does not invalidate the results obtained since the analyzed microRNAs were good biomarkers of response to treatment, especially miR-383-5p.

## 5. Conclusions

MiRNAs are important molecules in the understanding of tumorigenesis and resistance to therapy. Many characteristics have been discussed to establish good predictors of response to fg-SRL treatment in acromegaly. Our study has identified three miRNAs that are involved in the response to fg-SRL. miR-181a-5p and miR-181b-5p are biomarkers of response to fg-SRL treatment, and miR-383-5p is a biomarker of unresponsiveness. These miRNAs could be used as predictors of response individually or they could be included in prediction models to facilitate clinical decisions.

## Figures and Tables

**Table 1:** Characterization of controlled and uncontrolled patients.

	Controlled (*n* = 13)	Uncontrolled (*n* = 19)	*p*-Value
Median age (years)	41 (23–59)	33 (21–56)	0.05
Gender (male %)	38.5 (5/13)	63.1 (12/19)	0.15
Median pretreatment GH (ng/mL)	2.8 (0.7–15.8)	5.1 (0.5–35)	0.2
Median GH decrease (%)	88.6 (32.4–94.9)	34.0 (−523.2–86.0)	0.003
Median pretreatment IGF-I (xULN)	2.4 (1.3–3.0)	2.8 (1.3–4.0)	0.08
Median IGF-I decrease (%)	71.0 (51.5–81.1)	32.4 (−67.6–75.9)	0.001
Median largest tumor diameter (cm)	2.0 (1.0–6.1)	2.6 (1.0–4.7)	0.6
Invasive tumor (%)	25 (2/8)	61.5 (8/13)	0.18
Sparsely granulated (%)	25 (3/9)	62.5 (10/14)	0.1
Median SST2 (IRS)	12 (6–12)	7 (0–12)	0.06
Median SST5 (IRS)	4 (2–12)	9 (0–12)	0.6

GH: growth hormone; IGF-I: insulin-like growth factor type I; xULN: times the upper limit of normal; SST2: somatostatin receptor type 2; SST5: somatostatin receptor type 5. The Mann–Whitney U test and Fisher’s exact tests were performed for numeric and categorical variables, respectively. Numerical data are expressed as median (min–max). Categorical data are expressed as percentages of available data.

**Table 2:** Characterization of patients whose samples were used for miRNA sequencing.

	Controlled (*n* = 5)	Uncontrolled (*n* = 5)
Median age (years)	37.0 (35.0–59.0)	25.0 (21.0–33.0)
Gender (male %)	40.0 (2/5)	40.0 (2/5)
Median pretreatment GH (ng/mL)	1.8 (0.7–4.0)	8.0 (2.8–21.9)
Median GH decrease (%)	83.0 (32.4–94.1)	−18.7 (−523.2–17.8)
Median pretreatment IGF-I (xULN)	2.4 (1.7–2.7)	2.8 (1.8–3.7)
Median IGF-I decrease (%)	74.8 (65.8–81.1)	−41.9 (−67.6–42.8)
Median largest tumor diameter (cm)	2.0 (1.4–3.7)	2.4 (1.0–4.1)
Invasive tumor (%)	25.0 (1/4)	66.7 (2/3)
Sparsely granulated (%)	25.0 (1/4)	75.0 (3/4)
Median SST2 (IRS)	12 (6–12)	5 (0–12)
Median SST5 (IRS)	4 (4–12)	9 (0–12)

GH: growth hormone; IGF-I: insulin-like growth factor type I; xULN: times the upper limit of normal; SST2: somatostatin receptor type 2; SST5: somatostatin receptor type 5. Numerical data are expressed as median (min–max). Categorical data are expressed as percentages of available data.

**Table 3:** Characterization of patients used for biological validation by qPCR.

	Controlled (*n* = 8)	Uncontrolled (*n* = 14)	*p*-Value
Median age (years)	44.0 (23.0–58.0)	38.0 (22.0–56.0)	0.34
Gender (male %)	37.5 (3/8)	78.6 (11/14)	0.08
Median pretreatment GH (ng/mL)	2.8 (0.9–15.8)	3.7 (0.53–65.2)	0.64
Median GH decrease (%)	89.1 (44.6–94.9)	47.1 (−95.8–85.9)	0.002
Median pretreatment IGF-I (xULN)	2.4 (1.3–3.0)	2.8 (1.4–4.0)	0.19
Median IGF-I decrease (%)	68.6 (51.5–75.2)	34.9 (−8.8–75.9)	0.003
Median largest tumor diameter (cm)	2.6 (1.0–6.1)	2.6 (1.2–4.7)	0.66
Invasive tumor (%)	25.0 (1/4)	60.0 (6/10)	0.56
Sparsely granulated (%)	40.0 (2/5)	58.3 (7/12)	0.62
Median SST2 (IRS)	12 (6–12)	9 (4–12)	0.30
Median SST5 (IRS)	6 (2–12)	9 (0–12)	0.90

GH: growth hormone; IGF-I: insulin-like growth factor type I; xULN: times the upper limit of normal; SST2: somatostatin receptor type 2; SST5: somatostatin receptor type 5. Numerical data are expressed as median (min–max). Categorical data are expressed as percentages of available data.

**Table 4:** Median miRNA qPCR relative expression (minimum and maximum values) in each group.

miRNA	Controlled (*n* = 8)	Uncontrolled (*n* = 14)	*p*-Value
miR-10b-5p	2.67 × 10^−5^ (0–5.30 × 10^−3^)	1.76 × 10^−5^ (0–1.11 × 10^−3^)	0.595
miR-27b-3p	4.37 × 10^−2^ (7.19 × 10^−3^–1.19 × 10^−1^)	3.35 × 10^−2^ (4.19 × 10^−3^–1.14 × 10^−1^)	0.482
miR-181a-5p	1.13 × 10^−1^ (7.31 × 10^−3^–1.12)	2.75 × 10^−2^ (1.10 × 10^−3^–4.84)	0.075
miR-181b-5p	3.55 × 10^−2^ (3.42 × 10^−3^–2.94 × 10^−1^)	1.18 × 10^−2^ (2.33 × 10^−3^–6.20 × 10^−1^)	0.071
miR-199b-5p	2.00 × 10^−4^ (0–1.35 × 10^−2^)	3.60 × 10^−5^ (0–1.28 × 10^−2^)	0.185
miR-383-5p	1.73 × 10^−5^ (0–5.78 × 10^−3^)	2.97 × 10^−3^ (2.37 × 10^−5^–6.82 × 10^−2^)	0.041
miR-505-5p	8.20 × 10^−6^ (0–6.35 × 10^−4^)	9.08 × 10^−6^ (0–1.45 × 10^−3^)	0.773
miR-629-3p	1.59 × 10^−5^ (0–1.25 × 10^−4^)	2.62 × 10^−6^ (0–1.87 × 10^−3^)	0.862

## Data Availability

NGS data was deposited on Havard Dataverse (https://dataverse.harvard.edu/) at https://doi.org/10.7910/DVN/XVTSMO. Some datasets generated during and/or analyzed during the current study are not publicly available but are available from the corresponding author on reasonable request.

## Supplementary Materials

The following supporting information can be downloaded at: https://www.mdpi.com/article/10.3390/ijms24032875/s1.
