# Supplementary material for: miR-383-5p, miR-181a-5p, and miR-181b-5p as Predictors of Response to First-Generation Somatostatin Receptor Ligands in Acromegaly
Source: Int J Mol Sci. 2023 Feb 2;24(3):2875. doi: 10.3390/ijms24032875 (PMC9918086; doi:10.3390/ijms24032875)
Supplement: Supplementary file 1 [file ijms-24-02875-s001.zip › ijms-2132541-supplementary.pdf]

Supplemental material

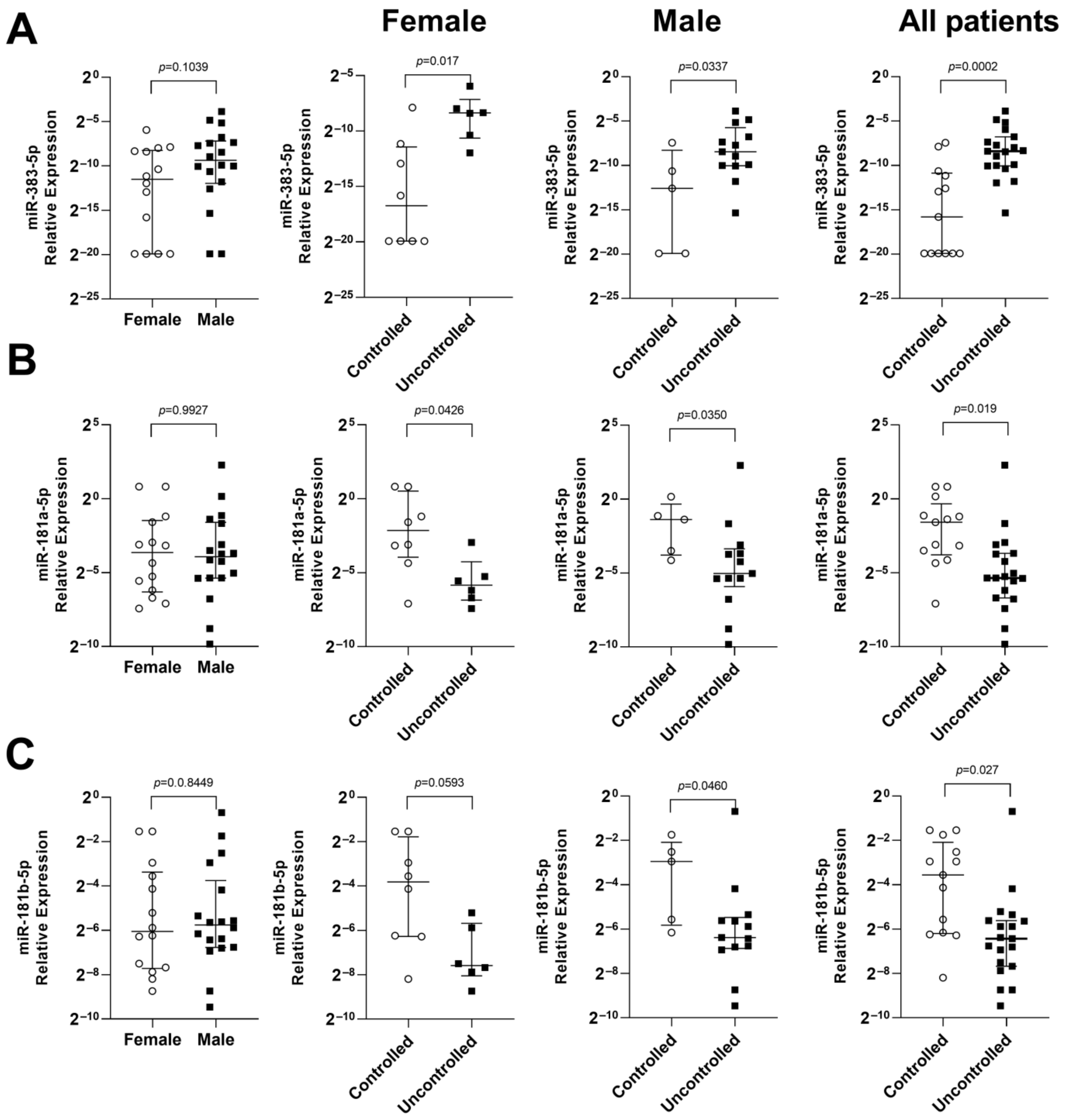

Supplementary Figure S1: Analysis of miRNA relative expression among males and females and response to treatment. Comparison of miR-383-5p (**A**), miR-181a-5p (**B**) and miR-181b-5p (**C**) between: male and females, controlled and uncontrolled females, controlled and uncontrolled males; controlled and uncontrolled patients.
